# Possibility of genome editing for melon breeding

**DOI:** 10.1270/jsbbs.23074

**Published:** 2024-04-09

**Authors:** Satoko Nonaka, Hiroshi Ezura

**Affiliations:** 1 Laboratory of Vegetable and Ornamental Horticulture, Institute of Life and Environmental Sciences and Tsukuba-Plant Innovation Research Center, University of Tsukuba, 1-1-1 Tennodai, Tsukuba, Ibaraki 305-8572, Japan

**Keywords:** melons, genome editing, breeding techniques

## Abstract

Genome editing technologies are promising for conventional mutagenesis breeding, which takes a long time to remove unnecessary mutations through backcrossing and create new lines because they directly modify the target genes of elite strains. In particular, this technology has advantages for traits caused by the loss of function. Many efforts have been made to utilize this technique to introduce valuable features into crops, including maize, soybeans, and tomatoes. Several genome-edited crops have already been commercialized in the US and Japan. Melons are an important vegetable crop worldwide, produced and used in various areas. Therefore, many breeding efforts have been made to improve its fruit quality, resistance to plant diseases, and stress tolerance. Quantitative trait loci (QTL) analysis was performed, and various genes related to important traits were identified. Recently, several studies have shown that the CRISPR/Cas9 system can be applied to melons, resulting in its possible utilization as a breeding technique. Focusing on two productivity-related traits, disease resistance, and fruit quality, this review introduces the progress in genetics, examples of melon breeding through genome editing, improvements required for breeding applications, and the possibilities of genome editing in melon breeding.

## Introduction

Melons (*Cucumis melo* L.) are annual herbaceous plants. Its main production areas range from the Mediterranean Region to East Asia. According to FAOSTAT statistics, in 2021, the global harvested area of melon was 1,077,369 ha, the production was 28,617,598.39 t, and the yield was 265,625 t/ha. It is a commercially important horticultural crop worldwide in terms of production quantity. Especially in Asia, melon is an important vegetable crop. Asia has the highest production, about 10 times higher than other areas, compared to Africa, America, and Europe, the Food and Agriculture Organization of the United Nations (FAO) (https://www.fao.org/faostat/en/#data/QC) (FAOSTAT 2021, Accessed on 01 November). Melons are used in various methods. In addition to being eaten as a dessert, some bitter or bland melons are consumed as vegetables. Due to its content of potassium, β-carotene, and vitamin C (ascorbic acid), the flesh of the melon fruit is considered a source of vitamins, minerals, and other health-promoting substances. Melon seeds are used as food in several countries, including India and Turkmenistan. Some melon fruits, such as small-fruited agrestis types and Waharman-type melons, are used after drying as preserved food when resources are scarce (Grumet *et al.* 2021). Melon can be widely utilized as food, thus improving its quality and productivity is essential, and a variety of new varieties have been developed.

Although melons have been classified into at least 19 horticultural subgroups and 6 groups (Pitrat 2016), there is little incompatibility or interbreeding between subgroups. Therefore, it can be said that melons have abundant genetic resources for breeding. Crossbreeding using DNA markers has been actively carried out using this rich genetic resource. Low incompatibility has accelerated the quantitative trait locus (QTL) analysis. Several QTL analyses with genetic diversity have provided practical DNA markers for important traits in breeding programs, such as improved fruit quality, plant disease resistance, and stress tolerance, and identified several associated genes. Selective breeding using DNA markers is a highly effective method, but it is not necessarily a panacea. The efficiency of this breeding method depends on the existing genetic diversity, which is limited in crops having experienced genetic bottlenecks during domestication. That is, if single nucleotide polymorphisms (SNPs) responsible for beneficial traits do not exist as genetic resources, selective breeding methods using DNA markers will not function satisfactorily. Mutagenesis and target-induced local lesioning (TILLING) platforms have been proposed as complementary techniques to complement this point (Dahmani-Mardas *et al.* 2010, Triques *et al.* 2007). This technology can introduce SNPs that did not exist in genetic resources. However, mutations may be presented simultaneously at multiple locations on the genome, and backcrossing is required to remove unnecessary mutations to develop cultivars. In this case, linkage dragging with the linkage of undesirable to desirable genetic traits should be considered. Although many excellent lines have been created by DNA maker selection and TILLING platforms, the limiting factors (genetic bottlenecks and linkage dragging) make conventional breeding a long process that can take, on average, a decade to develop a new plant variety. Expectations have been placed on genome editing as a new technology to accelerate breeding. This technology directly introduces mutations such as deletions and insertions into the target gene, so it does not introduce unnecessary mutations or chain resistance during the breeding process, and there is no need for backcrossing. Therefore, this technique can modify parental lines with excellent traits in the short term.

In genome editing technology, chimeric nucleases, such as zinc finger nucleases, transcription activator-like effector nucleases (TALENs), and clustered, regularly interspaced, short palindromic repeats (CRISPR)/CRISPR-associated protein 9 (CRISPR/Cas9), have been used as mutagens. CRISPR/Cas9 is relatively easier to design and create than the other two (Fichtner *et al.* 2014). However, it is said to have lower target specificity than the TALEN method. These nucleases induce double-strand breaks (DSBs) in specific genomic regions, which are then repaired. DSB repair can be broadly divided into homologous recombination (HR) repair, which uses a template to accurately repair, and non-homologous end joining (NHEJ), which involves error-prone repair. The DSB repair in plants frequently utilizes the non-homologous end joining (NHEJ) pathway (Fichtner *et al.* 2014). That is, the repair after cleavage of DNA on a target sequence by a chimeric nuclease involves insertion or deletion of bases. As a result, mutations are induced on the target sequence, resulting in loss or gain of gene function (first generation genome editing technology) (Giordano *et al.* 2022, Ito *et al.* 2015, Liu *et al.* 2022, Nizan *et al.* 2023, Nonaka *et al.* 2017). Several genome-edited crops have been commercialized in the United States and Japan, including tomatoes with high GABA content, waxy corn, and soybeans with high oleic acid content (Demorest *et al.* 2016, Ezura 2022, Gao *et al.* 2020, Ku and Ha 2020, Nonaka *et al.* 2017). Recently, base editing (targeted AID) (Bastet *et al.* 2019, Hunziker *et al.* 2020, Shimatani *et al.* 2017) and/or prime editing (Lin *et al.* 2020) have become available, allowing base substitutions. These technologies have made it possible to introduce SNPs (second generation genome editing technology). With the advent of second-generation genome editing technology, much more advanced genome editing is now possible, and it is becoming possible to meet the more detailed demands of breeding sites. For this reason, we can expect that the use of genome editing technology in breeding will further accelerate in the future.

For using genome editing technology as a breeding technology, it is necessary to obtain genome and genetic information. Genetic analysis of melon was performed and the genome size was estimated to be approximately 454 Mb based on nuclear DNA content (Arumuganathan and Earle 1991, Garcia-Mas *et al.* 2012). Whole genome sequences are already available through the useful databases Melonomics v4.0 (Ruggieri *et al.* 2018, https://www.melonomics.net) and CuGenDBv2 (Yu *et al.* 2023, http://cucurbitgenomics.org/v2/). When selecting target genes and regions using genome editing technology in breeding, not only information on gene function but also information on the site and timing of gene expression is important. A gene expression database is publicly available, and the site, timing, and intensity of gene expression are disclosed. (Yano *et al.* 2018, 2020, https://melonet-db.dna.affrc.go.jp/. For selection of CRISPR/Cas9 target sites and design guide RNA, databases (CRISPR-P 2.0, Liu *et al.* 2017, http://crispr.hzau.edu.cn/CRISPR2/ and/or CRISPR Plant, Minkenberg *et al.* 2019, http://omap.org/crispr//) are available.

In this way, tools to actually utilize genome editing technology in melon breeding are being developed. In this paper, we examine the possibility of breeding melons using genome editing technology. First, we will introduce research on gene function analysis to determine the most important target genes in genome editing technology. Many studies have provided information on loci and genes associated with important traits. In particular, we will introduce progress in functional genomics analysis of traits related to important traits targeted for melon breeding (productivity and fruit quality). Next, we will introduce an attempt to utilize the CRISPR/Cas9 system for melon breeding. The final section discusses improvements in genome editing techniques for breeding applications.

## Important traits for melon breeding

Traits related to productivity and quality, such as disease resistance, fruit quality, sugar content, aroma, nutrition, and fruit color, are the most important in melon breeding. Therefore, many studies have been conducted to identify the genes involved in these traits. This information is important for selecting target genes to introduce traits into melons via genome editing. This section introduces information on the genes or loci related to important traits for melon breeding.

### Disease resistance

Disease resistance traits are essential for increasing productivity, thus disease resistance is an important trait, and many efforts have been made to clarify the functions of resistance genes. Major diseases in melon are caused by viruses (33), fungi (10), and bacteria (3) (McCreight 2010–2011). We report on the research progress on resistance genes, using representative examples of several diseases. Genes introduced in this section are possible targets of genome editing.

#### Viral disease resistance

Viral diseases seriously damage melon productivity. Since viruses always parasitize cells, using chemicals to control the corresponding diseases is not an option. Therefore, the use of genetic resistance in breeding is the most reliable and cost-effective means to minimize losses. This review introduces results on resistance genes and loci for resistance of five viruses that cause the most significant economic losses on melons: Cucumber mosaic virus (CMV), Watermelon mosaic virus (WMV), Zucchini yellow mosaic virus (ZYMV), Papaya ringspot virus (PRSV), and Melon necrotic spot virus (MNSV). For a review of viral resistance causing other diseases, see Martín-Hernández and Picó (2021).

**CMV:** CMV causes typical mosaic symptoms on melon leaves, plant stunting, mottle or mosaic on fruits, and yield losses. This virus may have the widest host range of any known plant virus. Major QTL mapping has demonstrated that a single gene, cmv1, can confer complete recessive resistance to CMV subgroup II strains (Essafi *et al.* 2009, Guiu-Aragonés *et al.* 2015). At least two additional QTLs (cmvqw3.1 and cmvqw10.1) are required to confer resistance to subgroup I strains (Guiu-Aragonés *et al.* 2014). cmv1 encodes the vacuolar protein sorting 41 gene (*CmVPS41*). An amino acid residue (L348R or G85E) was identified as a polymorphism associated with a resistance phenotype (Giner *et al.* 2017, Pascual *et al.* 2019). These mutations alter the cellular localization of *CmVPS41*, restricting viral movement within bundle sheath cells and causing resistance to CMV (Pascual *et al.* 2019, Real *et al.* 2023).

**ZYMV:** ZYMV induces vein clearing and yellowing, blisters and enations on leaves, and severe stunting. On fruits, ZYMV induces mosaic or necrotic cracks, marbling, and hardening of the flesh. Moreover, isolates belonging to the pathotype F induce wilting in melons carrying the Fn gene (Risser *et al.* 1981) instead of mosaic in melons carrying the Fn+ allele. The Fn gene (for Flaccida necrosis) is present in numerous melon accessions. Three resistance candidate loci, namely *Zym-1*, *Zym-2*, and *Zym-3*, have been identified (Danin-Poleg *et al.* 1997, 2002, Díaz *et al.* 2003, Périn *et al.* 2002). More detailed analysis of the *Zym-1* locus has found *NBL-1* (*MELO3C015354*), *NBL-2* (*MELO3C015353*), and *NBL-3* with typical resistance genes of the coiled coil-nucleotide binding site of the leucine rich repeat family (CC-NB-LRR, abbreviated as NBL). Two NAC family transcription factors (*MELO3C015355* and *MELO3C015357*) are present in the adjacent region (Adler-Berke *et al.* 2021).

**WMV:** WMV induces mosaic, vein banding and deformation, such as blisters, filiformis and size reduction. It induces severe discoloration on fruits, with slight deformation in some cases. A major QTL for recessive resistance to WMV is located on chromosome 11 (Palomares-Ríus *et al.* 2011). Further analysis on chromosome 11 narrowed the locus of the resistance gene to a range of 141 kb. This locus was named *wmv1551*, and the SNP marker is known (Pérez-de-Castro *et al.* 2019). Three minor QTLs on chromosomes 4, 5, and 6 have been identified (Pérez-de-Castro *et al.* 2019).

**PRSV:** PRSV causes severe mosaicism, blisters, and malformations on leaves. Fruits may also show varying degrees of discoloration and deformity. Map-based cloning revealed that the PRSV resistance candidate gene (*Prv*) and the resistance candidate gene *Fom-1* of *Fusarium oxysporum* f.sp. melonis races 0 and 2 are located adjacent to each other on chromosome 11. Both of these genes encode proteins of the Toll/Interleukin-1 receptor domain intracellular nucleotide-binding leucine-rich repeat family (TIR-NLR) (Brotman *et al.* 2013). Because the two proteins (*Prv* and *Fom-1*) have similar structures, it was necessary to prove that the putative *Prv* is resistant to PRSV. To achieve this goal, mutations were introduced into *Prv* using the CRISPR/Cas9 system. *Prv* knockout mutant individuals showed susceptibility to PRSV. This revealed that *Prv* is a PRSV resistance gene (Nizan *et al.* 2023). An unusual nonconserved domain, encoding a second NBS domain was found at the C-terminus of Prv, which is likely to be related to PRSV resistance.

**MNSV:** MNSV induces small, greasy-looking to transparent spots on new leaves. On the fruit surface, it induces relatively large spots, resulting in a sunk and distorted appearance. Necrotic spots can be produced on stems. The recessive nsv resistance gene was identified (Coudriet *et al.* 1981), and hybrid melon cultivars carrying such resistance are widely used in commercial agriculture. nsv encodes Cm-elF4E, which is involved in translation initiation factors. One amino acid residue on *Cm-elF4E* (228Leu) showed resistance against all strains of MNSV except MNSV-264 (Nieto *et al.* 2006). An RNA sequence in the 3ʹ UTR of MNSV was essential for the resistance/recessive phenotype (Díaz *et al.* 2004). Using the CRISPR/Cas9-mediated cytosine base editor, two kinds of mutations were introduced on *Cm-elF4E*, *Cm-elF4E* (C322T/C323G) with a stop codon and *Cm-elF4E* (C322T/C323T, P108L) (Shirazi Parsa *et al.* 2023).

#### Viral disease transmitted by *Aphis gossypii*

Aphids are known pests that affect melon production. Heavy aphid colonization causes stunting, and severe leaf curling leads to plant death. Aphids excrete honeydew on the surface of leaves and fruits. This sticky, sweet substance allows the growth of sooty mold, which significantly reduces fruit quality. The aphid that colonizes melon crops is *A. gossypii*, which causes direct damage to melons and contributes to the spread of viral diseases as an efficient vector of viruses such as CMV, WMV, ZYMV, PRSV, and Cucurbitaceae-borne yellow virus (CABYV).

Entomologists, virologists, and plant geneticists have been working together to search for resistance genes in melons against aphid-mediated CMV infection. The Indian line PI 414723 and the Korean line PI 161375 show complete resistance to aphid-mediated CMV infection. In these lines, few aphids survived, and those that did survive had a low fecundity. These two phenotypes are thought to be co-segregated and controlled by a single sexual gene (Pitrat and Lecoq 1980, 1982). The genetic locus controlling this phenotype was named *Vat*, meaning “viral aphid transmission”. Aphid density is lower on Vat plants than on non-Vat plants (Thomas *et al.* 2016), this means Vat is involved in the resistance to aphid. Further genetic analysis revealed that the Vat locus encodes a 1467 amino acid protein belonging to the coiled-coil (CC)-nucleotide binding site (NBS)-leucine-rich repeat (LRR) family (Dogimont *et al.* 2014). An 11 kb DNA fragment containing the *Vat* resistance allele with a single coding sequence, native promoter, and terminator was transformed into an aphid-susceptible melon line (Vedrantais-Vat) and showed no response to the NM1 melon aphid clone. It showed high levels of resistance and complete resistance to the viruses upon inoculation with CMV, WMV, and ZYMV. Therefore, the 11 kb region containing the Vat gene seems to contain genes that function in both aphid resistance and virus resistance (Dogimont *et al.* 2014). To test this, resistance patterns to aphids and CMV were characterized by infection of nine aphid clones with PI 161375 and Vedrantais-Vat. In PI 161375, infection with eight aphid species showed CMV resistance, whereas in Vedrantais-Vat, infection with only three aphid species showed CMV resistance (Boissot *et al.* 2016a). These results indicate that at least additional loci are involved in CMV resistance induced by aphid infection in PI 161375. Accordingly, the *Vat* gene identified from PI 161375 was renamed *Vat-1*, and the presumed additional locus was named *Vat-2*. The genomic region spanning these genes is now called the Vat cluster (Boissot *et al.* 2016b). Five *Vat* homologs coding for proteins were identified in this cluster (Chovelon *et al.* 2021).

#### Resistance to other diseases

Powdery mildew (PM), Fusarium head blight (FW), gummy stem blight (GSB), and downy mildew (DM) are serious problem in melon production. these diseases are caused by fungi, oomycetes, and filamentous fung. Because these grow in high temperatures and humid conditions, these diseases are widespread in greenhouse. Most melon production are in greenhouse, thus the spread of these diseases leads to a decrease in melon production and causes economic losses. Therefore, controlling this disease is one of the important issues in melon breeding. Unlike viral diseases, some diseases caused by fungi, oomycetes, and molds can be controlled to some extent with fungicides. However, considering the negative effects of using fungicides on the environment and the human body, introducing resistance genes into melons is extremely effective. Therefore, introducing resistance to these pathogens into melons is essential for melon breeding. Abundant genetic resources have been analyzed, and many attempts have been made to search for resistance genes. This section introduces the loci, genes, and markers that have been discovered to confer resistance to these pathogens.

**PM:** Melon PM is often caused by *Podosphaera xanthii* (Px) and *Golovinomyces cichoracearum* (Gc) (Křístková *et al.* 2009, Li *et al.* 2017). *G. cichoracearum* occurs powderly in field environments in temperate and cold regions. *P. xanthii* is more frequently found in subtropical and tropical regions as well as in greenhouse crops (Natarajan *et al.* 2016, Ning *et al.* 2014). *P. xanthii* is emerging as the predominant pathogen in most countries (Bardin *et al.* 1999, Hosoya *et al.* 1999, Hudson *et al.* 2018, McCreight 2006, Ning *et al.* 2014, Pino *et al.* 2002, Zhang *et al.* 2012). Based on the reactions of isolates to melon differential lines, more than 28 physiological races of *P. xanthii* have been characterized (McCreight 2006). Due to race differentiation, resistant varieties are becoming susceptible to the disease in melons. It is necessary to breed varieties with multiple race-specific resistance genes and field resistance genes, and to date, several genes and QTL associated with resistance to PM have been mapped in different melon populations derived from several genetic sources (Table 1, Haonan *et al.* 2020). The identified QTLs have been listed by López-Martín *et al.* (2022).

Several candidates or resistance genes have been found from detailed analysis of chromosomes 5 and 12. The gene *MELO3C004311*, which encodes TMV resistance protein N-like, has been identified on chromosome 5 as a candidate resistance gene (López-Martín *et al.* 2022). Another *P. xanthii* resistance gene for races 1 and 3 (*Pm-w^WMR 29^*) has been identified in a cluster of nucleotide-binding leucine-rich repeat receptors (NLRs) on chromosome 5. Although *Pm-w^WMR 29^* is a homolog of the *A. gossypii* resistance gene *Vat-1*. *Pm-w^WMR 29^* does not confer resistance to aphids, and *Vat-1* does not confer resistance to PM (Boissot *et al.* 2024). The recessive resistance gene for race 2F “*Cmpmr2F*” (*MELO3C002403*), encoding allantoate amidohydrolase, was identified on chromosome 12 (Zhang *et al.* 2023a).

**FM:** FM is incited by the fungal pathogen *Fusarium oxysporum* Schlechtend f. sp. Melonis. Four physiological races of the pathogen (0, 1, 2, and 1.2) have been identified (Risser *et al.* 1976). *Fom-1* and *Fom-2* confer resistance to race 0 and race 2 and to race 0 and race 1, respectively. Fom-1 protein belongs to the TIR–NB–LRR type (Brotman *et al.* 2013). The putative Fom-2 protein includes the NB-ARC domain, an LRR-1domain, one Sfi1C (spindle body associated protein C-terminus) domain, and EAF (ELL-associated factor) domain (Wang *et al.* 2011a). These two genes have been utilized as commercial cultivars or as rootstocks. Race 1.2 can overcome these two resistance genes and include race 1.2y and 1.2w; race 1.2 yellowing (1.2y) induces yellowing symptoms before the death of the plants, and 1.2 wilting (1.2w) leads to wilting and death without the yellowing symptoms. Nine QTLs that provide resistance to race 1.2 have been found (Perchepied *et al.* 2005). Among the QTLs, *fomIII.1* and *fomVI.1* were specific for race 1.2y, while *fomV.2* and *fomXII.1* were only identified following inoculation with race 1.2w. Five QTL, *fomIII.2*, *fomIII.3*, *fomV.1*, *fomXI.1*, and *fomXII.1*, were effective against race 1.2y and race 1.2w. These results suggested that partial resistance to race 1.2 is governed by pathotype-shared loci and pathotype-specific loci. We screened 294 melon accessions, mainly of African and Asian origin, and found that the Indian accession PI124550 is resistant to both race 1.2y and race 1.2w. The highest resistance level was observed for both races in PI 124550, making it a promising breeding material with novel genes that confer useful resistance to both disease types in races 1.2 (Ishikawa *et al.* 2023). We look forward to future results regarding the discovery of resistance genes.

**DM:** Downy mildew (DM), caused by the obligate oomycete pathogen *Pseudoperonospora cubensis*, is a major foliar disease that causes great economic losses in melon production. Nine QTLs, including two major QTLs, for DM resistance were isolated from RILs (N = 169) generated from a cross between the resistant melon breeding line MR-1 and susceptible cultivar ‘Ananas Yok’neam’. The phenotype of the RILs was tested in both the greenhouse and growth chamber. Four QTLs (competitive allele-specific PCR [KASP] markers) were identified; of the major QTLs, *qPcub-10.1* was stable across the growth chamber and greenhouse tests, whereas *qPcub-8.2* was detected only in the growth chamber tests (Toporek *et al.* 2021, 2023). On chromosome 9, another QTL was identified from two F_2_ populations, which were bred from crossing the DM-resistant accession PI 442177 and two landraces, ‘Huangtu’ or ‘Huangdanzi’. Both QTL-seq and linkage map-based QTL mapping approaches have been used to identify a major QTL (DM9.1) associated with DM resistance (Zhang *et al.* 2023b).

**GSB:** GSB is a fatal fungus disease affecting most Cucurbit species, causing severe yield losses, especially in humid tropics and sub-tropics (Keinath *et al.* 1995, Zhang *et al.* 2017). In melon, the ascomycetous fungus, *Didymella bryoniae*. Rehm, is pathogenic. In the last sixty decades, although some GSB-resistance genetic loci for *D. bryoniae* have been reported in different resistant germplasm (Table 2), a functional GSB-resistant gene has not been identified.

The rapid development of molecular biology and sequencing technologies has accelerated the isolation of GSB-resistant genes. In the past 5 years, several genes controlling resistance to GSB have been identified. A single recessive resistance gene (*MELO3C022157*) was isolated on chromosome 9. *MELO3C022157* encodes a nucleotide-binding site leucine-rich repeat (BS-LRR), which is a resistance (R) genes. A comparison of the sequence of *MELO3C022157* between the resistant and susceptible lines indicated polymorphism, such as deletions in the first intron, a 2-bp frameshift deletion from the second exon, and a 7-bp insertion in the fourth exon of the resistant line. Based on this information, a molecular marker has been developed (Hassan *et al.* 2018). Another single dominant candidate resistance gene (*MELO03C012987*) was identified on chromosome 4, which encodes a protein similar to the uncharacterized Avr9/Cf-9 rapidly elicited (ACRE) protein 146 (Hu *et al.* 2018). A new single dominant resistance gene, MELO3C010403, has been isolated on chromosome 7. *MELO3C010403* encodes a protein with a wall-associated receptor-like kinase (WAK-RLK), critical for plant resistance to various pathogens; hence it is potentially associated with GSB resistance. Therefore, *MELO3C010403* is the most likely candidate gene (Ma *et al.* 2023).

### Sex-based expression

Sex-based expression in melon flowers is an important trait in melon breeding programs. Commercially available melon varieties usually have bisexual flowers. Monoecious plants, with female and male flowers, are of great value in melon breeding as they obviate emasculation to ensure hybrid purity. Therefore, many studies have been conducted, and the genes and mechanisms involved in sex determination have been clarified. Genes involved in the regulation of sex expression in melons are dominated by the expression of andromonoecious (*M*), androecious (*A*), and gynoecious (*G*) genes, which define the dioecious pathway (Boualem *et al.* 2015).

The *A* and *M* genes encode *CmACS11* and *CmACS7*, respectively, two aminocyclopropane-1-carboxylic acids (ACC) synthase (ACS) enzymes, which catalyze the rate-limiting step of ethylene production in the plant (Boualem *et al.* 2008, 2015). The *G* gene encodes a C_2_H_2_ zinc finger transcription factor of the transcription factor WIP protein subfamily (*CmWIP1*) (Martin *et al.* 2009). The expression of *CmACS11* suppresses the expression of *CmWIP1* (Boualem *et al.* 2015). *CmWIP1* suppresses the expression of the carpel formation gene *CRABS CLAW* (*CmCRC*), inhibits differentiation into female flowers, and promotes male flower formation (Zhang *et al.* 2022). Ethylene generated by *CmACS7* mediates a signal transduction system and controls the expression of the homoeodomain class I transcription factor HD-ZIP I, *CmHB40*, suppressing stamen formation in hermaphrodite flowers and suppressing female flower formation (Rashid *et al.* 2023).

Recently, four promising candidate genes related to sexual expression in oriental melon were identified on Chr. 1 and 8, namely *MELO3C015898* (transport inhibitor response 1), *MELO3C015904* (SWR1 complex protein 4/DNA methyltransferase 1-associated protein 1), *MELO3C024563* (putative UDP-N-acetylglucosamine-peptide N-acetylglucosaminyltransferase SPINDLY), and *MELO3C024565* (MRNA decapping enzyme-like protein) (Kishor *et al.* 2021).

QTLs, *tpbf2.1*, *Opbf3.1*, and *tpbf8.1* have been found on chromosomes 2, 3, and 8, respectively. *Opbf3.1* and *tpbf8.1* promote the development of pistil and stamen primordia by inhibiting the functions of *CmWIP1* and *CmACS-7*, respectively, indicating the possibility of inducing hermaphroditism (Nashiki *et al.* 2023).

Sexual expression in developing flower buds is plastic, and it has been suggested that environmental factors influence sexual expression. A heterochromatin protein (*CmLHP1*) affects sex determination, and the relationship with epigenetic regulation is becoming clearer (Rodriguez-Granados *et al.* 2022).

### Fruit trait

Melon fruit, an economically and agriculturally important plant, shows huge diversity in pericarp and fruit flesh color, nutrition, sugar content, aroma, and ripening style. These traits are deeply involved in fruit quality and are among the most important traits for breeding. Therefore, several studies have been conducted that are related to fruit traits such as the color of fruit flesh, sugar content, aroma, and ripening. Many QTLs have been listed in the review by Shahwar *et al.* (2023). Here, we introduce the identified gene-related fruit traits.

**Fruit color:** Two genes (*CmOr* and *CmFBN1*) are involved in fresh, orange-colored fruit. There are three colors: orange, green, and white. The orange color is caused by the accumulation of β-carotenoid, a precursor of vitamin A, and its synthesis is controlled by the *CmOr* gene (Chayut *et al.* 2017, Tzuri *et al.* 2015). Carotenoid accumulation is regulated by the carotenoid sequestration protein FIBRILLIN1 (*CmFBN1*) (Zhou *et al.* 2023). A comparison of proteomic analysis of a high β-carotene melon variety and its isogenic line with a defective *CmOr* revealed impaired chromoplast formation and identified *CmFBN1* as differentially expressed. *CmFBN1* enhances *CmOR*-triggered carotenoid accumulation by stimulating plastid globule proliferation for carotenoid sequestration within chromoplasts.

**Sugar content:** Several related genes have been identified, including sucrose phosphate synthase (*CmSPS1*), acidic invertase (*CmAI*) (Hubbard *et al.* 1989), and sucrose transporters (*CmTST1-3*) (Cheng *et al.* 2018). QTL analysis with ‘PS’ and ‘SC’ showed three important loci that were related to sugar content on chromosomes 4, 5, and 7 (Argyris *et al.* 2017).

**Aroma:** Eighty-two volatile organic compounds (VOCs) biosynthesized in melon rind and flesh were detected by gas chromatography-mass spectrometry (GC-MS). An RIL population from the cross ‘PS’ × ‘VED’ showed 166 QTLs and a major QTL cluster was identified on chromosome 8, which was the same as the ripening-related QTL *ETHQV8.1*. QTLs related to esters, lipid-derived volatiles, and apocarotenoids were identified. Candidate genes have been proposed for ethyl 3-(methylthio) propanoate and benzaldehyde biosynthesis (Mayobre *et al.* 2021). A set of Near-Isogenic Lines (NILs) containing overlapping introgressions from the Korean accession ‘SC’ showed climacteric ripening QTL, *ETHQB3.5*, were also involved in VOCs biosynthesis (*e.g.*, 3-methylbutyl acetate, benzyl acetate, 2-methylbutan-1-ol, 2-methylpropyl acetate, and 1-methylsulfanylbutan-1-one) (Zhao *et al.* 2023).

**Ripening:** Melon employs two types of ripening systems: climacteric (ethylene-dependent) and non-climacteric (ethylene-independent) ripening, depending on the horticultural subgroups. Other fruits do not include two systems, usually within the same species; only a single system is used (for example, tomato is only a climacteric type, and strawberries are only non-climacteric). Therefore, melon is a good source-material for the genetic analysis of climacteric and non-climacteric species. In climacteric fruits, ethylene triggers multiple responses (*i.e.*, changes in texture, fruit firmness, expression of cell wall-degrading enzymes, fruit color, and aroma). Genetic analyses were performed to clarify the mechanism of ethylene evolution. Three major QTLs (*ETHQB3.5*, *ETHQV6.3*, and *ETHQ8.1*) were found in the NILs derived from the non-climacteric melon parental lines ‘SC’ and ‘PS’ and population of RILs, obtained by crossing a climacteric ‘VED’ and a non-climacteric variety ‘PS’ (Pereira *et al.* 2020, Vegas *et al.* 2013). *ETHQV6.3* includes *CmNOR* related to the ethylene signaling pathway (Liu *et al.* 2022), and *ETHQ8.1*, which contains *CmCTR* involved in epigenetic control and *CmROS1*, which is a transcription factor (Giordano *et al.* 2022). Using the CRISPR/Cas9 system, we confirmed that these genes are key for controlling climacteric ripening. The knockout of *CmNOR* using the CRISPR/Cas9 system delays ripening in the climacteric type of melon (Liu *et al.* 2022). The destruction of *CmCTR* and *CmROS* in climacteric melons results in earlier ethylene production. This result indicates that the loss-of-function of these genes accelerates the ripening stage in climacteric fruits (Giordano *et al.* 2022).

**Shelf-life:** A long shelf-life is a crucial trait in fruit crop breeding because it directly affects food loss and waste. Globally, 14% of the total food production is lost before retail sales (English *et al.* 2019), and an additional 17% is wasted at the retail and consumer levels (Forbes *et al.* 2021), resulting in a staggering loss of USD 400 billion. These losses are especially significant for fruits and vegetables because of their poor shelf-lives compared to cereals and non-perishable goods (English *et al.* 2019). Therefore, the ability to modify plants to improve their shelf-life should improve the sustainability of the global food system by reducing food loss and waste. Moreover, a long shelf-life is crucial for exporting fruits and vegetables, as maintaining fruit quality and freshness requires close control of temperature, humidity, oxygen, and carbon dioxide, leading to high energy usage and costs. Therefore, producing a parental line with a long shelf-life can reduce the energy and costs required for export (Lamberty and Kreyenschmidt 2022).

Ethylene, a gaseous plant hormone, is a key regulator of fruit shelf-life. Ethylene is synthesized from the amino acid methionine and first converted into S-adenosyl-L-methionine (SAM) by SAM synthase. SAM is the primary methyl donor in plants and is involved in the methylation of lipids, proteins, and nucleic acids. SAM is converted by aminocyclopropanecarboxylate (ACC) synthase into 5-methylthioadenosine, which is converted back into methionine and then into ACC, the precursor of ethylene. ACC is oxidized by ACC oxidase (ACO) into ethylene. Therefore, ACO is a key enzyme involved in the regulation of ethylene production. In many plant species, the *ACO* gene has several homologs with highly conserved sequences. Melonet-DB (https://melonet-db.dna.affrc.go.jp/ap/top) shows that the melon genome contains five *CmACOs*, and the gene *CmACO1* is predominantly expressed in harvested fruits (Yano *et al.* 2020). Previous studies have shown that suppressing the *ACO* gene via antisense or RNA-i genetic modifications extends fruit shelf-life by 5–14 d in climacteric melons (Ayub *et al.* 1996, Nuñez-Palenius *et al.* 2006). Substitutional mutation of *ACO* via ethyl methanesulfonate (EMS) treatment and TILLING selection also extended the shelf-life of melon (Dahmani-Mardas *et al.* 2010). The TILLING study showed that the G194D mutation resulted in longer shelf-life in melon fruit (Dahmani-Mardas *et al.* 2010). Based on these results, *CmACO1* is expected to be a key gene for the shelf-life of melons.

## Case study of application of genome editing technology in breeding research: Improved shelf-life by CRSPR/Cas9

Fruits and vegetables produced in Japan are highly regarded for their extremely high quality, safety, and reliability, and demand is gradually increasing. Introducing long-lasting traits is important for promoting exports, including further expanding sales channels. Japanese melon varieties are known for their high quality and are popular overseas. However, problems persisted, including the short shelf life and high transportation costs for exportation. It has been shown that *CmACO1* is one of the key genes to control shelf-life through reverse genetic analysis with anti-sense and EMS technology. To breed a new melon lien with a long shelf-life, mutations should be introduced only in *CmACO1* into existing melon varieties without affecting other genes. Antisense and RNAi technologies that have been used to date are based on transformation technologies, and crops produced using these technologies are classified as genetically modified crops (GM crops). GM crops are strictly regulated and require safety evaluation. Mutagenesis by EMS is also used as a breeding technique. Backcrossing is required to introduce a mutation into a single gene in an existing variety due to the simultaneous introduction of mutations into genes other than the target genes by this technique. The original genome set may be lost during backcrossing due to effects such as linkage drag. Crops produced by genome editing can be considered non-GM crops because genome editing technology does not leave foreign genes in the final crop. Additionally, since modifying only the target gene is possible, the breeding period is expected to be considerably shorter than conventional breeding methods such as mutation breeding. Recently, using genome editing technology, we succeeded in modifying *CmACO1* to ‘Earl’s Favourite’, which is used as the parent variety line of Japanese high-grade melon (*C. melo* var. ‘reticulatus’). Modification of *CmACO1* suppressed ethylene production from fruit. Wild-type and genome-edited melons were stored at room temperature for 14 days, and the storage periods were compared. In the wild type, fruit softened on the 14th day of storage, the epidermis collapsed, and deterioration of the fruit was observed. In contrast, in the genome-edited plants, the fruit remained firm even after 14 days of storage and did not deteriorate. Based on the above, we succeeded in introducing a long shelf-life trait by modifying his *CmACO1* using genome editing technology (Nonaka *et al.* 2023).

## Improvements in genome editing technology for breeding applications

For genome editing for crop breeding, genomic information is essential for select target genome. Beside, stable techniques are also important for introducing the CRISPR/Cas9 system into crop genome. In melons, genomic information with important traits has been made available through considerable efforts (see “Important traits for melon breeding”). Moreover, few genetic databases have been made available, such as Melonomics v4.0 (Ruggieri *et al.* 2018, https://www.melonomics.net), CuGenDBv2 database (http://cucurbitgenomics.org/v2/), and Melonet DB (Yano *et al.* 2018, 2020, https://melonet-db.dna.affrc.go.jp/ap/top).

In a recent study that used a CRISPR/Cas9 system in melons, 40–100% of transgenic lines with CRISPR/Cas9 showed a mutation in the target gene, these result meanst that the occurrence of mutations was high with CRISPR/Cas9 system (Giordano *et al.* 2022, Nonaka *et al.* 2023). Generally, to introduce the CRISPR/Cas9 system into crop genome, Agrobacterium-mediated transformation technology is used. Although several transformation protocols have been reported for melons, the transformation frequency is usually quite low and depends on the cultivar. Additionally, melons tends to become tetraploid in tissue culture with high frequency. To avoid these problems, tissue culture systems such as selection and redifferentiation and genome editing might be done without tissue culture, such as the iPB method (Imai *et al.* 2020).

AAs discussed in “Important traits for melon breeding”, many useful traits, such as disease resistance, fruit traits, and sexual expression, are often due to SNPs rather than genetic defects. Therefore, in breeding, genome editing technology that can induce SNPs is more practical than the simple CRISPR/Cas9 system that induces gene disruption. Single nucleotide substitution enzymes (base editor/target-AID) that can replace C to T, G to A, A to G, and T to C, have developed and can be used in rice and tomatoes. (Bastet *et al.* 2019, Hunziker *et al.* 2020, Shimatani *et al.* 2017). Regarding the targeted AID method, it can also be used in melon, where base and amino acid substitutions were successfully made in the MNSV resistance gene *Cm-elF4E* (Shirazi Parsa *et al.* 2023). Furthermore, prime editing, which specifies the location of the target site and replaces the target DNA nucleotide by linking reverse transcriptase to nCas9, has been developed and can be used in rice and wheat (Lin *et al.* 2020). CRISPR/Cas9, base editor/target-AID, and prime editing utilize the Cas9 protein. Due to the Cas9 protein, the end of the target sequence (PAM sequence) must be NGG. This point is the only factor that limits the selection of target sequences. The development of Cas proteins that can recognize various PAM sequences is progressing, and there will be almost no restrictions on target arrays.

Genome editing technology is advancing rapidly and is becoming an easy-to-use technology for breeding new varieties. From a simple technical perspective, there is great potential to accelerate the development of new varieties. However, there are some hurdles that must be overcome for commercial use, such as patent issues. In order to use it as a breeding technology and commercially utilize new varieties, there is a need to develop new technologies that keep patent fees low, as well as technology transfer systems, packaging, and consulting that make it easier to utilize existing genome editing technologies. becomes important. Widespread use also requires expanding public understanding of this technology.

## Author Contribution Statement

This manuscript was mainly written by S.N. S.N. and H.E. planned and refined the overall structure of the text.

## Figures and Tables

**Table 1.** Identifried PM resistnce loci

Chromosome/Linkage group	*Loci*	Ref.
Chr02	*Pm-x*	Périn *et al.* 2002
	*Pm-x1.5*, *Pm-x3*	Fazza *et al.* 2013
	*Pm2F*	Zhang *et al.* 2013
	*QTL* (*AR5*)	Fukino *et al.* 2008
	*Pm-Edisto47-2*	Ning *et al.* 2014
Chr04	*qPx1-4*	Branham *et al.* 2021
Chr05	*Pm-w*	Pitrat 1991
	*Pm-R1-2*	Yuste-Lisbona *et al.* 2011
	*PM-R5*	Yuste-Lisbona *et al.* 2011
	*Pm-AN*	Wang *et al.* 2011b
	*qPx1-5*	Branham *et al.* 2021
	*QTL PmV-1-Pi124112*	Perchepied *et al.* 2005
Chr10	*CmPMrs*	Cui *et al.* 2022
	*qPx1-10*	Branham *et al.* 2021
Chr12	*Pm-y*	Périn *et al.* 2002
	*QTL PmXII-1-Pi124112*	Perchepied *et al.* 2005
	*QTL* (*AR5*)	Fukino *et al.* 2008
	*Pm-Edisto47-1*	Ning *et al.* 2014
	*CmPMR1*	Cui *et al.* 2022
	*Cmpmr2F*	Zhang *et al.* 2023a
	*qPx1-12*	Branham *et al.* 2021
	*qCmPMR-12*	Cao *et al.* 2021
	*BPm12.1*	Li *et al.* 2017
LGIX	*Pm-1*	Teixeira *et al.* 2008

**Table 2.** Identifried GBS resistnce loci

*Loci*	*Hereiary type*	Plant Intrduction accession	Ref.
Gsb-1	Dominance	PI140471	Sowell *et al.* 1966
			Frantz and Jahn 2004
Gsb-2	Dominance	PI157082	Zhang *et al.* 1997
			Frantz and Jahn 2004
Gsb-3	Dominance	PI511890	Zhang *et al.* 1997
			Frantz and Jahn 2004
Gsb-4	Dominance	PI482398	Zhang *et al.* 1997
			Frantz and Jahn 2004
Gsb-5	Recessive	PI420145	Frantz and Jahn 2004
Gsb-6	Dominance	PI482399	Zhang *et al.* 1997
